# Invagination of the Sphenoid Sinus Mucosa after Endoscopic Endonasal Transsphenoidal Approach and Its Significance

**DOI:** 10.1371/journal.pone.0162836

**Published:** 2016-09-13

**Authors:** Do Hyun Kim, Yong-Kil Hong, Sin-Soo Jeun, Jae-Sung Park, Ki Hwan Jung, Soo Whan Kim, Jin Hee Cho, Yong Jin Park, Yun Jin Kang, Sung Won Kim

**Affiliations:** 1 Department of Otolaryngology-Head and Neck Surgery, Seoul St. Mary’s Hospital, College of Medicine, The Catholic University of Korea, Seoul, Korea; 2 Department of Neurosurgery, Seoul St. Mary’s Hospital, College of Medicine, The Catholic University of Korea, Seoul, Korea; Universita degli Studi di Palermo, ITALY

## Abstract

**Objective:**

To describe the clinical features of invagination of the sphenoid sinus mucosa (ISM) and compare them with other similar cases using a visual analog scale (VAS) to assess the various nasal symptoms and to discuss its clinical significance and means of prevention.

**Study Design:**

Retrospective chart review at a tertiary referral center.

**Methods:**

Between 2010 and 2015, 8 patients who had undergone EETSA surgery displayed postoperative ISM. The comparison group consisted of 147 patients who underwent the same surgical procedures and were diagnosed with the same diseases. Pre- or postoperative paranasal sinus computed tomography (PNS CT) and VAS were performed and subsequently analyzed.

**Results:**

The clinical features of ISM of the sphenoid sinus showed sellar floor invagination and regenerated inverted ingrowing sphenoid mucosa on endoscopic imaging. PNS CT also showed a bony defect and invaginated air densities at the sellar turcica. Pre- and postoperative VAS scores revealed that the ISM group had much less of an improvement in headaches after surgery than that of the comparison group (*p* = 0.049).

**Conclusion:**

ISM may occur because of a change in pressure, sphenoid mucosal status, or arachnoid membrane status. Moreover, ISM is related to improvements in headaches. Therefore, EETSA patients should avoid activities that cause rapid pressure changes during the healing process. In addition, sellar reconstruction that is resistant to physical pressure changes should be mandated despite the absence of an intraoperative CSF leak.

## Introduction

The suprasellar extension descended into the pituitary fossa with inversion of the tumor dome and of the diaphragma sellae could be occurred after the removal of a pituitary adenoma[1]. The pattern of descent of the diaphragma sellae to the sella was variously reported. Tumor volume, diabetes insipidus, residual tumor, or CSF leak could affect the descent pattern[2]. After the removal of a pituitary macroadenoma, sphenoid sinus mucosa was regenerated. Sphenoid sinus mucosa repositioning methods provide a good basis for postoperative healing[3,4] and insufficient mucosa regeneration could have been reported the cause of such a delayed CSF leak[3]. However, even after performing sphenoid sinus mucosa reposition, we experienced 8 cases of invagination of sphenoid sinus mucosa (ISM) after endoscopic endonasal transsphenoidal approach (EETSA) surgery during the previous 6-year period.

In this report, we describe the clinical features of ISM of the sphenoid sinus and compare them with other similar cases using a visual analog scale (VAS) to assess the various nasal symptoms. We then discuss the clinical meaning, significance, and prevention of ISM with sellar floor reconstruction using hard materials.

## Methods

**Fig 1:**
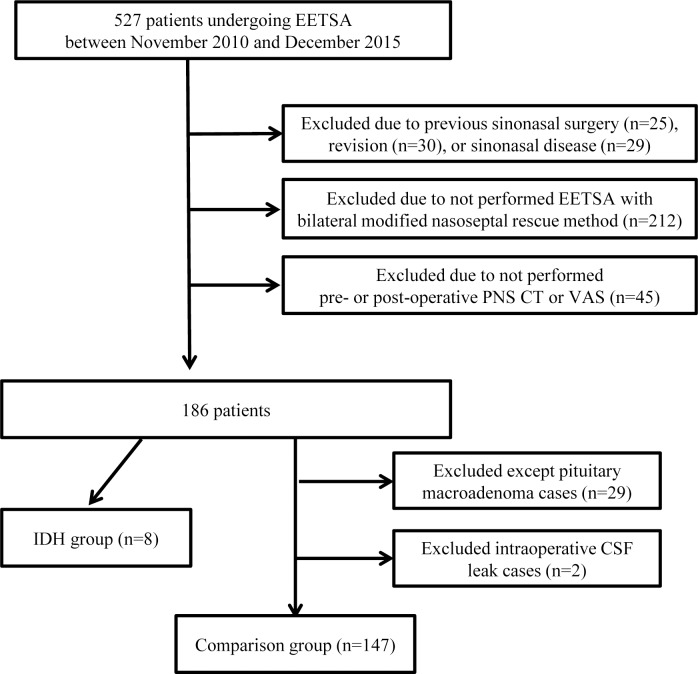
Flow chart of the study design.

This study and the retrospective chart review were approved by the institutional review board of Seoul St. Mary's Hospital (KC16RISI0422). Our Institutional Review Board approval waived the need for informed consent for this retrospective chart review. Between November 2010 and December 2015, 527 patients with sellar and parasellar skull base tumors underwent surgery via EETSA. Patients with a history of previous sinonasal surgery, revision, sinonasal diseases, or patients who did not undergo pre- or postoperative paranasal sinus computed tomography (PNS CT) and VAS were excluded. We also excluded patients who did not undergo EETSA with bilateral modified nasoseptal rescue flaps[5]. As described in detail previously[5], the inferior, middle, and superior turbinates were preserved and lateralized, and the posterior bony septum that included a portion of the perpendicular plate of the ethmoid bone, the vomer, and the anterior wall of the sphenoidal sinus was removed. The removed posterior bony septum was preserved in saline as a prevention against CSF leaks. Septoplasty was not performed, and the most posteriorly located ethmoid air cell was removed to allow wider sphenoid exposure[5]. Eight patients showed postoperative ISM and did not meet the exclusion criteria. These patients were diagnosed as pituitary macroadenoma, and reconstruction materials such as autologous nasal septal bone were not used, because there was no confirmed CSF leak as determined by the Valsalava maneuver. In addition, all 8 cases had undergone repositioning of the sphenoid mucosa. We therefore created a comparison group limited to pituitary macroadenoma patients who did not experience an intraoperative CSF leak, in whom reconstruction materials were not used, and who did not meet the exclusion criteria. Cases that did not undergo posterior sphenoid mucosa repositioning were excluded from the group. Therefore, 372 patients had been excluded and the comparison group was comprised of 147 patients (Fig 1).

**Fig 2:**
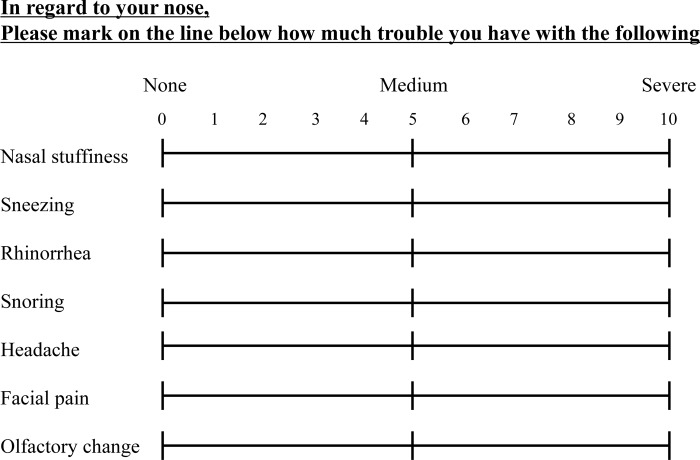
Visual analog scale.

Patients underwent pre- and postoperative PNS CT and VAS. PNS CT was performed with previously reported setting[6]. The tumor volume was calculated according to the following formula: tumor volume = 4/3π x (height/2 x width/2 x length/2)[7–10]. The VAS consisted of nasal stuffiness, sneezing, rhinorrhea, snoring, headache, facial pain, and olfactory changes and were scored subjectively (each score ranged from 0 to 10; Fig 2). Higher scores indicated more severe nasal symptoms. Posterior sphenoid sinus wall endoscopic images were obtained using a 4-mm, 0° nasal endoscope (Olympus Corp., Tokyo, Japan). Postoperative PNS CT, VAS scores, and images were obtained after 6 months.

All data are expressed as means ± standard deviation. The Mann-Whitney test was used to compare differences in pre- and postoperative changes in the VAS score between the ISM of the sphenoid sinus group and the comparison group. A *p*-value < 0.05 was considered to indicate statistical significance. All statistical analyses were conducted using the SAS software (ver. 9.3; SAS Institute, Cary, NC, USA).

## Results

The mean patient age was 49.3 years (range, 30–69 years) in the ISM group; 4 patients (50%) were males, and 4 (50%) were females. The comparison group comprised 147 patients, 87 (59.1%) males and 60 (40.8%) females, with a mean age of 49.4 years (range, 17–80 years). There were no significant differences between two groups (Table 1).

**Table 1:** Study population.

	ISM group (n = 8)	Comparison group (n = 147)	p-value
Sex (M:F)	4: 4	87: 60	0.609
Age	49.3±15.93	49.4±14.12	0.974

### Clinical features of ISM of the sphenoid sinus

**Fig 3:**
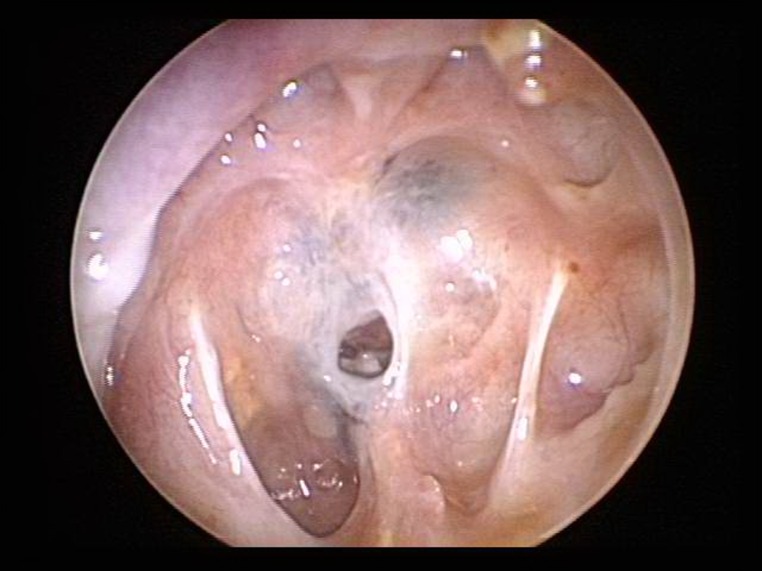
Postoperative endoscopic image of the posterior sphenoid sinus wall 6 months after surgery.

**Fig 4:**
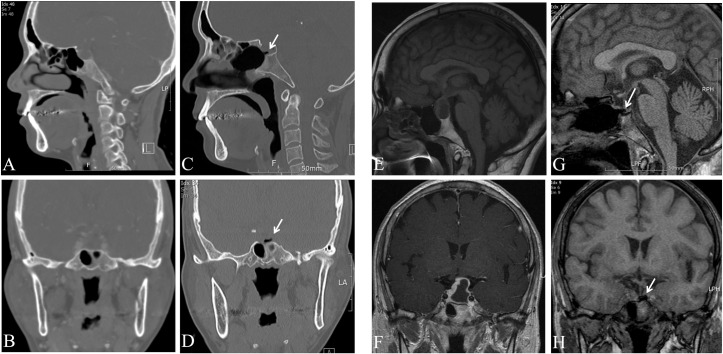
Pre- and post-operartive images of patient showing invagination of the sphenoid sinus mucosa. (A,B) Pre-operartive PNS CT; sagittal and coronal setting. (C,D) Post-operartive PNS CT; sagittal and coronal setting. (E,F) Pre-operartive PNS MRI T1; sagittal and coronal setting. (G,H) Post-operartive PNS T1; sagittal and coronal setting. Arrows indicate invaginated lesion of the sphenoid sinus mucosa.

**Fig 5:**
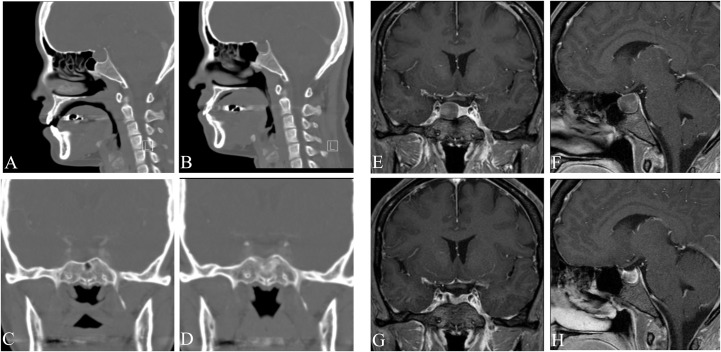
Pre- and post-operartive images of patient in the comparison group. (A,B) Pre-operartive PNS CT; sagittal and coronal setting. (C,D) Post-operartive PNS CT; sagittal and coronal setting. (E,F) Pre-operartive PNS MRI T1; sagittal and coronal setting. (G,H) Post-operartive PNS T1; sagittal and coronal setting.

Fig 3 shows postoperative 6 months endoscopic images of the sellar floor, and Figs 4 and 5 shows pre- and post-operative PNS CT and PNS magnetic resonance imaging (MRI) images 6 months after surgery in both groups. Endoscopic images revealed sellar floor invagination and regenerated inverted sphenoid mucosa with an ingrowing status. The PNS CT sagittal section also showed a bony defect and invaginated air densities at the sellar turcica.

### Correlation of pre-operative tumor volume and ISM occurrence

Table 2 shows pre-operative tumor volume between two groups. There were no significant pre-operative tumor volume differences between the ISM and comparison groups (*p* = 0.891).

**Table 2:** Correlation of pre-operative tumor volume and ISM occurrence.

	ISM group (n = 8)		Comparison group (n = 147)		p-value
Tumor size	Mean (mm3)	SD	Mean (mm3)	SD	
	7134.86	8600.61	6524.61	7870.85	0.891

### Difference in changes in nasal symptoms between the ISM and comparison groups

Pre- and postoperative VAS scores are presented in Table 3. Nasal stuffiness (*p* = 0.993), sneezing (*p* = 0.848), rhinorrhea (*p* = 0.864), snoring (*p* = 0.709), facial pain (*p* = 0.360), and olfactory changes (*p* = 0.531) did not show significant changes between the ISM and comparison groups. However, the headache score showed significant changes between the groups. The ISM group exhibited a reduced improvement in headaches after surgery (*p* = 0.049).

**Table 3:** Differences between the pre- and postoperative visual analog scale.

	ISM group (n = 8)		Comparison group (n = 147)		p-value
	Pre-Post		Pre-Post		
VAS items	Mean	SD	Mean	SD	
Nasal stuffiness	0.250	2.435	0.095	2.330	0.993
Sneezing	0.625	2.387	0.170	2.365	0.848
Rhinorrhea	0.250	1.909	-0.007	2.451	0.864
Snoring	0.875	2.357	0.497	2.856	0.709
Headache	-0.250	1.282	1.810	2.927	0.049*
Facial pain	1.000	1.852	0.235	2.547	0.360
Olfactory change	-1.125	2.588	-1.918	3.459	0.531

*P<0.05 for the test.

ISM, invagination of sphenoid sinus mucosa; SD, standard deviation.

## Discussion

One of the most common complications after endoscopic endonasal transsphenoidal surgery for anterocentral skull base tumors is the leakage of intraoperative cerebrospinal fluid (CSF) after removal of the tumor. Following the transsphenoidal approach, if an intraoperative CSF leak is absent, sellar reconstruction by autologous or synthetic materials or by pedicled nasoseptal flaps is not performed routinely[11–13]. However, when an intraoperative CSF leak is discovered, it is necessary to perform sellar reconstruction using hard materials[12,13]. As a result of this, a variety of reconstruction materials and surgical methods using autologous nasal septal cartilage, bone and sphenoid sinus bone, or various synthetic materials such as titanium, alumina ceramic, stainless steel, silicone, and bio-absorbable mesh have been developed[12–19].

After surgery, the sphenoid sinus becomes mucosalized. The sellar floor diaphragm normally bulges slightly toward the sphenoid because of intracranial pressure or maintains a smooth continuous margin along the posterior sphenoid wall by balancing the mucosal healing process and intracranial pressure. However, if, during the sellar flow diaphragm healing process, patients sneeze succrently, blow their nose, or defecate, ISM might occur. A thin arachnoid membrane exposed after removal of the tumor with dura might, in several cases, cause an ISM. In addition, an incomplete unfolded sphenoid mucosa during repositioning might also result in ISM. Once ISM occurs, sellar floor diaphragm sagging might occur due to changes in the pressure of the nasal cavity. Therefore, the performance of physical or leisure activities that cause rapid pressure changes, such as scuba diving or sky diving, or particular occupations such as pilots, would be restricted in ISM patients even after mucosal healing. Among our 8 ISM patients, 2 complained of headache aggravation, and 1 patient experienced different headache symptoms such as dizziness. Analysis of the pre- and postoperative VAS scores revealed that patients in the ISM group did not experience a significant improvement in headaches compared with the comparison group. This suggested the clinical significance of ISM.

Therefore, to prevent ISM, sellar reconstruction using materials resistant to physical pressure change is required regardless of the absence of an intraoperative CSF leak. Also, Locatelli et al[20]. reported sellar floor reconstruction with hard material in all cases promotes a more rapid and complete healing and helps recovery of mucociliary function in the sphenoid sinus. However, pedicled nasoseptal flaps play a limited role in physical pressure changes and donor site morbidity. Thus, autologous tissue such as posterior nasal septal bone, which can be harvested easily during EETSA, or synthetic materials would be useful and valid candidates.

Our study has strength in that the comparison group underwent the same surgical procedures and were diagnosed with the same diseases as the ISM group, thus allowing the groups to be well matched. However, some limitations were also apparent. The sample size of 8 cases provides a relatively small group for the comparison of results. Thus, this sample size differences might represent a significant bias. VAS scores were only followed up once, 6 months after surgery. The retrospective nature of the work renders the findings weaker than those afforded by randomized controlled studies. Therefore, future studies should include a large number of patients over a longer follow-up period.

## Conclusion

ISM might arise from changes to the intracranial and nasal cavity pressure. Moreover, ISM is related to an improvement in headaches. Therefore, EETSA patients should refrain from blowing their nose and abstain from physical activities that cause rapid pressure changes during healing of the sellar floor. In addition, sellar reconstruction materials should be reinforced using materials resistant to physical pressure changes despite the absence of an intraoperative CSF leak.
